# A New Approach to Detect Mover Position in Linear Motors Using Magnetic Sensors

**DOI:** 10.3390/s151026694

**Published:** 2015-10-21

**Authors:** Sarbajit Paul, Junghwan Chang

**Affiliations:** Mechatronics Laboratory, Department of Electrical Engineering, Dong-A University, 840, Hadan-2-dong, Saha-gu, Busan 604-714, Korea; E-Mail: pol.jit@gmail.com

**Keywords:** Hall Effect, linear motor, magnetic sensor, position detection

## Abstract

A new method to detect the mover position of a linear motor is proposed in this paper. This method employs a simple cheap Hall Effect sensor-based magnetic sensor unit to detect the mover position of the linear motor. With the movement of the linear motor, Hall Effect sensor modules electrically separated 120° along with the idea of three phase balanced condition (*v_a_ + v_b_ + v_c_* = 0) are used to produce three phase signals. The amplitude of the sensor output voltage signals are adjusted to unit amplitude to minimize the amplitude errors. With the unit amplitude signals three to two phase transformation is done to reduce the three multiples of harmonic components. The final output thus obtained is converted to position data by the use of arctangent function. The measurement accuracy of the new method is analyzed by experiments and compared with the conventional two phase method. Using the same number of sensor modules as the conventional two phase method, the proposed method gives more accurate position information compared to the conventional system where sensors are separated by 90° electrical angles.

## 1. Introduction

Recently, linear motors have been used in many areas of manufacturing and automatic transfer system for flat panel display and factory automation. With the increase in the application of linear motors, the position estimation method has captured a lot of attention. In conventional methods, linear encoders are used as the most common position sensors [1,2,3]. A linear encoder gives precise position information, but for a long track application, the use of linear encoders is very expensive and difficult to implement due to the size of the encoder. Many researchers have also used sensorless algorithms to control the permanent magnet motors. In the case of linear motors sensorless position estimation methods were also used [4,5,6]. They can be divided into two categories: back EMF method [7,8,9,10] and signal injection method [11,12], although under low speed conditions, the back EMF method cannot offer good performance. In contrast, the signal injection method works well in the low speed area but suffers from saliency problems and cross saturation [13].

To achieve an alternative low cost and robust method for position detection, magnetic sensors are widely used nowadays. Linear position sensors with an accuracy of 0.2 mm to 1 mm can be designed using Hall Effect sensors [14]. Hall Effect position sensors are contactless, with long lifetime in industrial atmospheres and are of low cost. Hall Effect sensors are grouped into Discrete Hall Effect sensors and Linear Hall Effect sensors. Discrete Hall Effect sensors are widely used in BLDC motors [15,16,17]. Three Hall Effect sensors separated 120° electrically are used to produce three square waves. However, the rough resolution of 60° limits the application of Discrete Hall Effect sensors. An improved method to estimate the angular position in brushless permanent magnet machines is proposed in [18]. Discrete Hall Effect sensors were also used in [19,20] to estimate the position of linear motors. In Linear Hall Effect sensors, the output signal is continuous in nature. They are widely used in PMSM control applications. In [21], Linear Hall Effect sensors are introduced for the angular measurement application. The use of Linear Hall Effect sensors for linear position measurement was proposed in [22]. As to the application of magnetic position sensors, in conventional two phase system, two position sensors placed 90° apart from each other are employed for position detection. In conventional three phase systems, three position sensors electrically displaced 120° are used to decode the mover position [23,24,25]. Appropriate mounting position of the linear Hall Effect sensor improves the accuracy of sensor output [26]. Thus the mounting position and the proper distance between Hall Effect sensors can provide improved information about the mover position in linear motors.

The concept of the present research is motivated by the distinguishing characteristics between conventional two phase and three phase systems for position detection. The work presented in this paper has a couple of steps to precisely achieve mover position: firstly, the conventional magnetic sensor position detection methods using conventional two phase and three phase systems were investigated and compared under equal amount of errors in amplitude and phase. If the amplitudes of the sensor signals are made a unit reference, the three phase system yields better position results than the two phase system. In addition to this, if three to two phase transformation is done on three phase system signals, the resulting two phase signal shows a reduction of multiple of three order harmonics. 

Secondly, considering the advantages of the conventional three phase system over two phase systems mentioned above, a new position detection method for accurate position detection of linear motors using Hall Effect-based magnetic sensors is proposed. The new position detection method uses same number of sensors like a conventional two phase system, but gives superior result compared to the conventional two phase system. The proposed position detection method produces three phase signals using two magnetic sensor units electrically separated 120° and three phase balanced condition (*v_a_ + v_b_ + v_c_* = 0). Three phase signal is made to two phase signal using three to two phase transformation. The position of the linear motor is achieved by using an arctangent function on the two phase signal obtained. The significance and performance of the proposed method is verified by MATLAB/Simulink. In addition, the experimental verification is also achieved by using magnetic sensor units. The proposed method does not need any extra error compensation methods to minimize the errors individually. 

Section 2 describes the basic principle of the position detection using the conventional position sensing methods. In Section 3, a comparison between the conventional methods of position detection is presented with a MATLAB simulation model. Section 4 explains the idea of the proposed method and its simulation results. The overview of the magnetic position sensor used to detect the position of the mover is presented in Section 5. Finally in Section 6 experimental analysis and the validity of the proposed method are discussed.

## 2. The Principle of Position Detection

A conventional two phase magnetic sensor system [23,24] consists of two sensing units electrically displaced 90° to produce two orthogonal signals. Position is obtained by using an arctangent function on the outputs of the sensors. For an ideal two phase sensing system, the outputs of the sensing units will be sine and cosine signals expressed as follows:

(1) $$\begin{array}{l} u_{a}=U\sin\theta \\ u_{b}=U\cos\theta \end{array}$$

Taking arctangent to the equation, position can be measured as:

(2) $$\theta =\arctan\left(\frac{u_{a}}{u_{b}}\right)$$

(3) $$x=\frac{\tau_{p}}{\pi }\theta$$

where *τ_p_* is the pole pitch of the motor.

In a three phase system [25], the sensors are electrically displaced 120°. A three to two phase transformation is done on the outputs of the sensor units to get two phase voltages *v_α_*, *v_β_*. By the use of the arctangent function the position detection is achieved. Mathematically, the three phase signal is represented as:

(4) $$\begin{array}{l} v_{a}=V\sin\theta \\ v_{b}=V\sin\left(\theta -\frac{2\pi }{3}\right)\\ v_{c}=V\sin\left(\theta +\frac{2\pi }{3}\right) \end{array}$$

Three to two phase transformation results in:

(5) $$\begin{array}{l} v_{\alpha }=\sqrt{\frac{2}{3}}\left(v_{a}-\frac{v_{b}}{2}-\frac{v_{c}}{2}\right)\\ v_{\beta }=\sqrt{\frac{2}{3}}\left(\frac{\sqrt{3}v_{b}}{2}-\frac{\sqrt{3}v_{c}}{2}\right) \end{array}$$

Position can be measured as follows:

(6) $$\theta =\arctan\left(\frac{v_{\alpha }}{v_{\beta }}\right)$$

(7) $$x=\frac{\tau_{p}}{\pi }\theta$$

## 3. Analysis of the Conventional Position Methods

To analyze two conventional position detection systems for linear motors, an equal amount of errors in amplitude, phase and harmonics are considered on the outputs of the sensor units and their influences on the sensor systems are then checked.

### 3.1. Error in Amplitude 

Let us first consider only amplitude error for both the systems. For a two phase system, Equation (1) can be rewritten as:

(8) $$\begin{array}{l} u_{a}=U_{a}\sin\theta \\ u_{b}=U_{b}\cos\theta \end{array}$$

where *U_a_* and *U_b_* is the amplitude for both the signals. 

Similarly for the three phase system, considering the error in amplitude only, Equation (4) is rewritten as:

(9) $$\begin{array}{l} v_{a}=V_{a}\sin\theta \\ v_{b}=V_{b}\sin\left(\theta -\frac{2\pi }{3}\right)\\ v_{c}=V_{c}\sin\left(\theta +\frac{2\pi }{3}\right) \end{array}$$

Taking the three phase to two phase transformation of Equation (9):

(10) $$v_{\alpha }=\sqrt{\frac{2}{3}}\left(v_{a}-\frac{v_{b}}{2}-\frac{v_{c}}{2}\right)=\left(\frac{\sqrt{6}}{3}V_{a}+\frac{\sqrt{6}}{12}(V_{b}+V_{c})\right)\sin\theta +\frac{\sqrt{2}}{4}\left(V_{b}-V_{c}\right)\cos\theta$$

(11) $$v_{\beta }=-\frac{\sqrt{2}}{4}(V_{b}-V_{c})\sin\theta -\frac{\sqrt{6}}{4}(V_{b}+V_{c})\cos\theta$$

**Figure 1:**
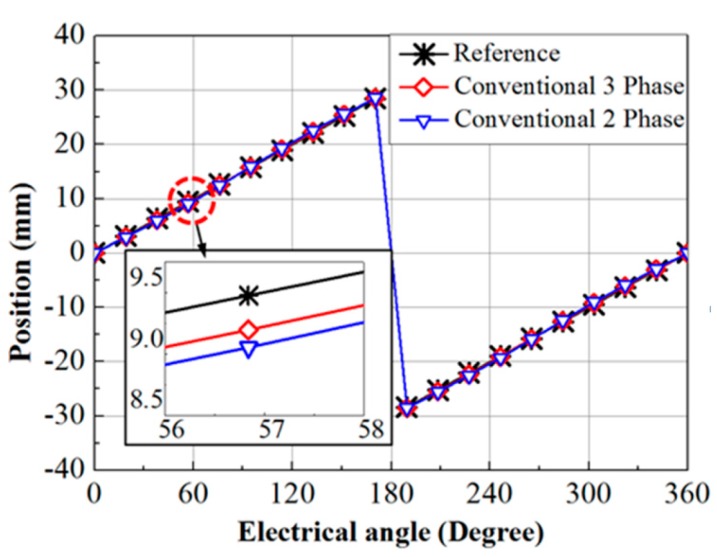
Simulations of the position calculation for conventional two phase and three phase sensing systems under equal amount of error in amplitude.

MATLAB simulation models for two phase and three phase position detection systems are made to observe the deviation of the output signal under 1% error in amplitude for both the conventional systems. The simulation result is shown in Figure 1. The simulation of the position detected by the conventional three phase system has an average error of 2.9% in position compared to the ideal reference signal, whereas the position data obtained from the conventional two phase system has an average position error of 5.5% in the position in comparison to the ideal reference signal. From Equations (10) and (11), if amplitudes are made equal, *v_α_* and *v_β_* will be perfect sine and cosine signals, so in the proposed method, the amplitudes of measured sensor signals are made to unit amplitude to get perfect sine and cosine signals.

### 3.2. Error in Phase

Similar to the amplitude error, the error in phase is considered individually for both the conventional two phase and three phase position detection systems as follows:

In case of a two phase system:

(12) $$\begin{array}{l} u_{a}=U\sin(\theta -\alpha )\\ u_{b}=U\cos(\theta -\alpha ) \end{array}$$

For the three phase system:

(13) $$\begin{array}{l} v_{a}=V\sin\theta \\ v_{b}=V\sin\left(\theta -\frac{2\pi }{3}\pm \alpha \right)\\ v_{c}=V\sin\left(\theta +\frac{2\pi }{3}\pm \beta \right) \end{array}$$

(14) $$\begin{array}{l} v_{\alpha }=\frac{V}{3}\left(2\sin\theta +\sin\left(\frac{2\theta \pm \alpha \pm \beta }{2}\right)\cos\left(\frac{\pm \alpha \mp \beta }{2}\right)-1.74\cos\left(\frac{2\theta \pm \alpha \pm \beta }{2}\right)\cos\left(\frac{\pm \alpha \mp \beta }{2}\right)\right)\\ v_{\beta }=\frac{\sqrt{3}V}{3}\left(\cos\left(\frac{2\theta \pm \alpha \pm \beta }{2}\right)\sin\left(\frac{\pm \alpha \mp \beta }{2}\right)+1.74\cos\left(\frac{2\theta \pm \alpha \pm \beta }{2}\right)\cos\left(\frac{\pm \alpha \mp \beta }{2}\right)\right) \end{array}$$

**Figure 2:**
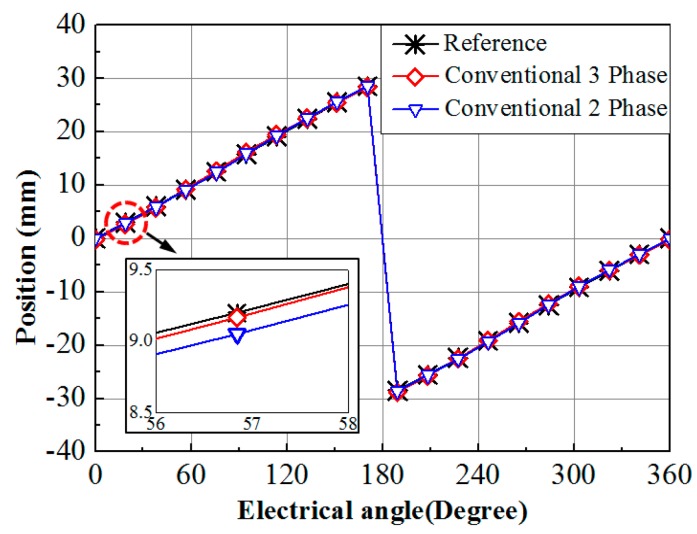
Simulations of the position calculation for conventional two phase and three phase sensing systems under equal amount of error in phase.

To check the phase error condition, MATLAB simulation with 1% error in phase for both conventional three phase and two phase system is considered. The variation of the position detected by both the systems and their deviation from the reference under phase error is illustrated in Figure 2. The deviation of the position signal from the reference signal is very small in both the systems. From simulation, the average errors in position are 0.85% for the conventional two phase system and 0.34% for the conventional three phase system compared to the reference position signal, respectively. 

### 3.3. Harmonic Components

Finally the effect of harmonics on both the systems is investigated. Let us assume the three phase system has third harmonic in Equation (15). During three to two phase transformation, the third harmonic disappear in the quadrature signals *v_α_* and *v_β_* obtained as a result of the transformation, shown in Equation (16). Similarly harmonics of 3’s multiples (9th, 15th, …) also disappear in the transformation process:

(15) $$\begin{array}{l} v_{a}=V_{a}\sin\theta +V^{\prime }\sin3\theta \\ v_{b}=V_{b}\sin\left(\theta -\frac{2\pi }{3}\right)+V^{\prime }\sin3\left(\theta -\frac{2\pi }{3}\right)=V_{b}\sin\left(\theta -\frac{2\pi }{3}\right)+V^{\prime }\sin3\theta \\ v_{c}=V_{c}\sin\left(\theta +\frac{2\pi }{3}\right)+V^{\prime }\sin3\left(\theta +\frac{2\pi }{3}\right)=V_{c}\sin\left(\theta +\frac{2\pi }{3}\right)+V^{\prime }\sin3\theta \end{array}$$

(16) $$\begin{array}{l} v_{\alpha }=\sqrt{\frac{2}{3}}\left(v_{a}-\frac{v_{b}}{2}-\frac{v_{c}}{2}\right)\\ \;=\left(\frac{\sqrt{6}}{3}V_{a}+\frac{\sqrt{6}}{12}(V_{b}+V_{c})\right)\sin\theta +\frac{\sqrt{2}}{4}(V_{b}-V_{c})\cos\theta \\ v_{\beta }=-\frac{\sqrt{2}}{4}(V_{b}-V_{c})\sin\theta -\frac{\sqrt{6}}{4}(V_{b}+V_{c})\cos\theta \end{array}$$

## 4. Proposed Method of Position Measurement

### 4.1. Principle of Proposed Method

It is found that when the amplitudes of the three phase signal output *V_a_*, *V_b_*, *V_c_* are made to unit amplitude and three to two phase transformation is carried out on three phase system which ultimately eliminates the third harmonic components, the three phase system gives better results compared to the two phase system. Therefore considering the above results, in this paper a method different from both the conventional methods to detect the position of linear motor is proposed. The proposed method presented in this work, consists of two sensing units electrically separated 120° instead of 90° to produce *v_a_* and *v_b_*, the two phase voltages of three phase system. To obtain the third voltage *v_c_*, three phase balanced condition is applied. The amplitudes are made to unit amplitude references to fulfill the minimum amplitude error condition mentioned in Section 3. Then three phase to two phase transformation is done and arctangent function is used to get the position.

### 4.2. Simulation Result

To test the validity of the proposed method, a MATLAB simulation model of the sensor module is made. The MATLAB simulated data obtained from the first two sensing modules are used to obtain the third voltage *v_c_*, using the three phase balanced condition. Three phase to two phase transformation is done followed by the arctangent function to get the position information.

The simulation result obtained is compared with a conventional two phase system under the same error conditions as that of the proposed model and it is found that the proposed system gives better position detection results compared to the two phase system under same condition.

**Figure 3:**
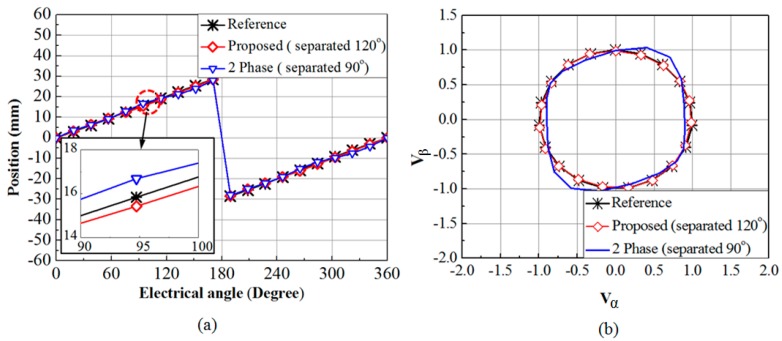
(**a**) Simulation of the position calculations for the proposed and conventional two phase sensing system (**b**) Lissajous curves for the proposed system and conventional two phase system.

Figure 3a shows the simulation result of the position obtained by proposed system and conventional two phase system with their deviation from the reference signal. Figure 3b illustrates the Lissajous curve for the proposed system and conventional two phase system. The position from the proposed system has a standard deviation of 0.018 mm from the reference and the conventional two phase system has a standard deviation of 0.701 mm from the reference. The simulation model of the proposed system gives better result compared to the two phase system.

## 5. Overview of Magnetic Position Sensor

To check the validity of the simulation results obtained in Section 4, experimental verification is done on the linear motor using Hall Effect-based magnetic sensor modules as discussed below. Position transducers based on Hall Effect sensors are widely used in automotive and industrial applications because of their low cost and long lifetime. The sensors use a physical phenomenon discovered by Edwin H. Hall [27] and named after him. The basic principle underlying the Hall Effect is Lorentz force. According to Hall's experiment when electric current is passed through a conductive material with magnetic field perpendicular to the plane of the conductor, a voltage difference appears across the conductor. If a current *I* flows through a conductor thickness *z* placed in a magnetic field of flux density *B*, hall voltage is given as:

(17) $$V_{H}=\frac{K_{h}BI}{z}$$

**Figure 4:**
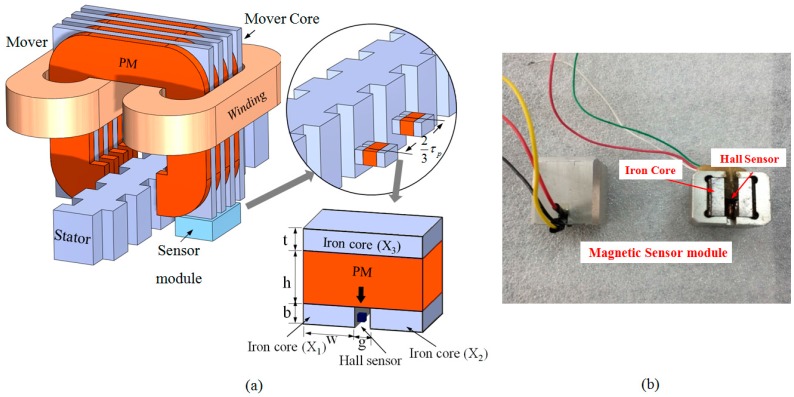
(**a**) Graphical representation of the arrangement of the sensor units over stator to obtain signal *v_a_* and *v_b_*; (**b**) manufactured magnetic sensor module.

The structure of the sensor module [28] and its arrangement over the linear motor to verify the proposed method is shown in Figure 4a. It is designed considering the application for transverse flux linear motor (TFLM) in which the moving direction of the mover is parallel to the current flow unlike the conventional longitudinal machines. The mover of the TFLM is composed of two windings, magnets (PMs) and mover cores. The PMs are magnetized along the moving direction with alternate polarity and are placed in between two adjacent mover cores to form high magnetic flux [29].

The magnetic sensor unit consists of a PM, made of Nd-Fe-B and three other parts X_1_, X_2_, X_3_ of silicon steel are attached with the magnet. Prototype of the sensor module is shown in Figure 4b. A Hall sensor is placed between X_1_ and X_2_. The Hall sensor used to design the sensor module is A1324 linear Hall Effect sensor manufactured by Allegro Microsystem LLC (Worcester, MA, USA). If the sensor unit moves parallel to the stator’s alternate tooth slot arrangement, a sinusoidal wave signal is generated, because when the sensor moves over the stator, the reluctance path of the magnetic fluxes from the sensor’s permanent magnet changes. As shown in Figure 4a, two sensor units are placed at a mechanical distance of 2/3*τ_p_* to produce sine waves of 120° phase difference. In Table 1, the specifications of the sensor module variables are listed.

**Table 1:** Specifications of the Hall sensor module variables.

Variables	Values (mm)	Variables	Values (mm)
t	3.0	w	7.0
h	7.0	g	2.0
b	2.8		

**Figure 5:**
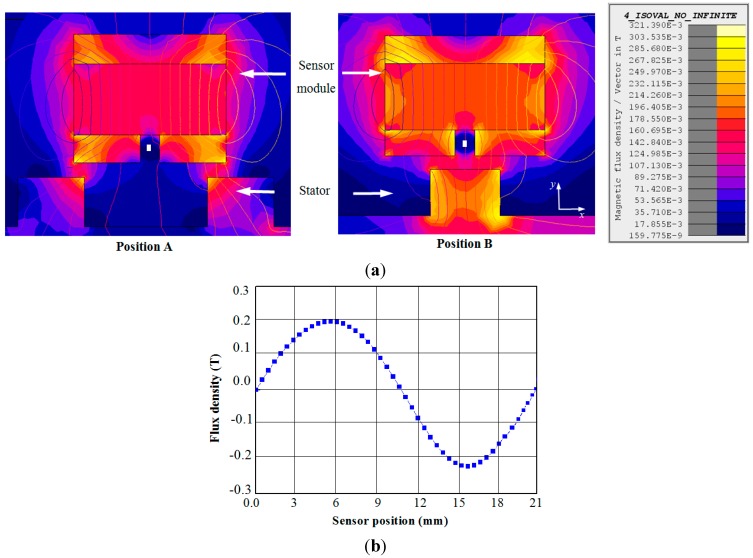
Two different positions of the magnetic sensor module (**a**) Position A: in between two teeth of the stator, Position B: aligned with the stator tooth and the flux flow path at A and B positions respectively; (**b**) change in flux density according to the position of the magnetic sensor module.

**Figure 6:**
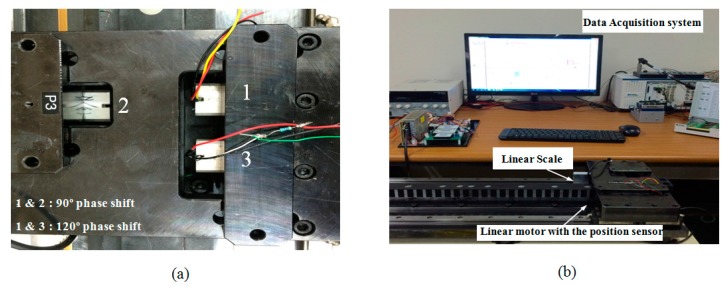
(**a**) Sensor module arrangement for position detection; (**b**) Experimental setup for the position measurement.

Figure 5a presents two position conditions of the magnetic sensor unit. The position of the magnetic sensor unit in between two stator teeth is represented by position A and position B represents the moment when sensor module is aligned with the stator tooth. Finite element analysis (FEA) of the sensor module is done using Flux 2D software and the variation of the leakage flux of the sensor module at two distinct positions A and B are shown in Figure 5a. From Figure 5a, it is clear that at position A and B there is symmetry in the flux distribution. As a result of this, the flux distributions in *x* direction become zero at A and B respectively. Thus for two pole pitch displacement of the sensor module over the stator, the flux density distribution becomes sinusoidal as shown in Figure 5b. Figure 6 shows the arrangement of the sensor modules with the linear motor and the experimental setup respectively. The specification of the stator used for the experiment and the PM for the sensor is given in the Table 2.

**Table 2:** Specification of the experimental device.

Items	Parameters	Values
Stator	Material	50PN600 *
	Pole Pitch, *τ*_p_	10 mm
	Tooth height, T_h_	5 mm
	Tooth Width, T_w_	7 mm
	Slot width, T_s_	13 mm
Permanent Magnet	Material	NdFeB **
	Residual Magnetic Flux Density	1.2 T
	Relative permeability	1.05

* Non oriented electrical steel with thickness 0.5 mm, core loss is smaller than 6 W/kg at 50 Hz, Magnetic flux density is greater than 1.66 T at 5000 A/m; ** Neodymium magnet.

## 6. Experimental Verification and Results 

**Figure 7:**
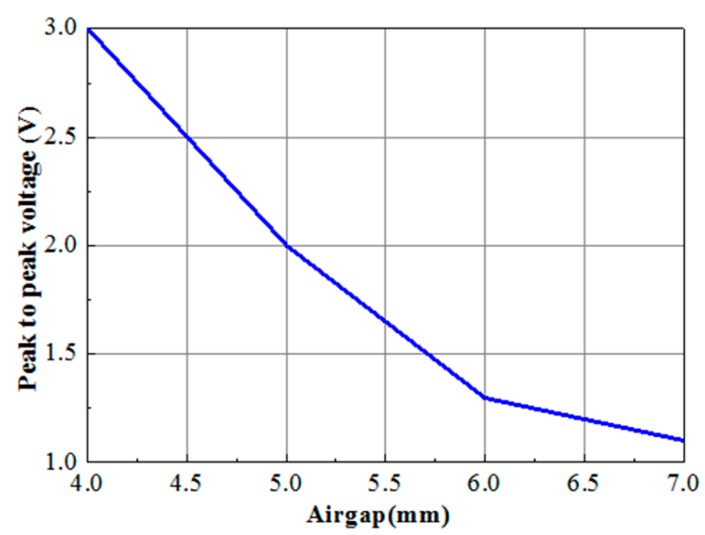
Peak to peak value of the sensor output signal with the variation of airgap.

To test the effect of the variation of airgap between the stator and the sensor unit on the output sensor signal, the airgap is varied in 1 mm steps. The change in the peak to peak value of the output voltage signal with the variation of airgap is shown in Figure 7. The sinusoidal output voltage signal with maximum peak to peak value can be achieved with an airgap distance of 4 mm. If the airgap is further decreased, a sinusoidal waveform cannot be obtained. For further analysis, the airgap distance is fixed at 4 mm. At 4 mm airgap the peak to peak of the output voltage signal is 3 V.

**Figure 8:**
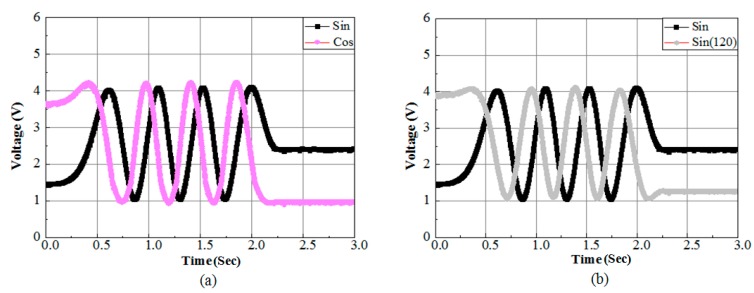
(**a**) Two phase (90° phase shift) and (**b**) proposed method (120° phase shift) sensor output.

Three sensor modules are used to produce two sets of signals. The first set of signals is phase shifted by 90° and the second set of signals has a phase shift of 120°. The sensor unit outputs are presented in Figure 8. Figure 8a shows the conventional two phase system outputs, phase shifted by 90°. The output data in Figure 8b shows the signals *v_a_* and *v_b_* of the proposed method, phase shifted by 120°. To achieve the third signal, *v_c_*, three phase balanced condition (*v_a_* + *v_b_* + *v_c_* = 0 or *v_c_* = − (*v_a_* + *v_b_*)) is applied.

**Figure 9:**
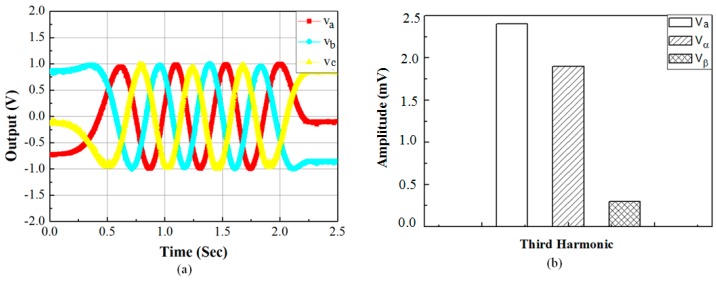
(**a**) Three phase signal obtained from proposed method; (**b**) Third harmonic components before and after three to two phase transformation.

The final three phase signal is shown in Figure 9a. As shown in Figure 9a, the amplitude of the three signals are made to unit amplitudes to minimize the error in amplitude. Three to two phase transformation is done on the final three phase signals to achieve the quadrature signals *v_α_* and *v_β_*. The FFT analysis of the three phase signals, *v_α_* and *v_β_* is carried out. FFT analysis shows that the amplitude of the 3rd harmonic component of the three phase signal is 0.0024 V. In three to two phase transformed signals *v_α_* and *v_β_*, third harmonics are reduced to 0.0019 V and 0.0003 V, respectively, shown in Figure 9b. Thus, three to two phase transformation has significantly reduced the 3rd order harmonics. This experimental observation is in accordance with the effect of three to two phase transformation on three multiple of harmonics mentioned before in Section 3.

**Figure 10:**
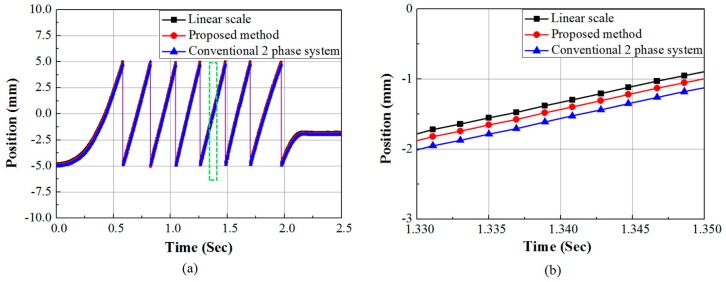
(**a**) Mover position using the proposed method and conventional two phase system; (**b**) Zoom in time and position axes of (**a**) to show the deviation of proposed and conventional two phase system output from reference position.

Finally to obtain position information an arctangent function is applied to both the conventional two phase system and the proposed system. The position data obtained from both the systems is compared with the reference position data obtained by using a linear scale. The position signals are shown in Figure 10a. The position information obtained using proposed system has a standard deviation of 1.40 mm from the reference linear scale signal, whereas the two phase system position signal shows a deviation of 2.16 mm from the reference. Figure 10b shows the zoom in waveforms within the broken line portion of Figure 10a. The deviation of the two signals from the reference is clearly seen in Figure 10b. Figure 10 confirms that the proposed method gives better mover position of the linear motor compared to the conventional two phase system. 

## 7. Conclusions

In this study, we have introduced a novel mover position detection method for linear motors, different from the conventional position detection approaches. The position is obtained by using two magnetic sensor modules mounted parallel to the stator and three phase balanced condition. Unlike the conventional methods, the proposed method does not use any complex compensation algorithm to minimize the error. Unit amplitudes of the sensor signals and three to two phase transformation are used to minimize the errors caused by amplitude difference and harmonics. The validity of the proposed method is checked using MATLAB simulation model and experimental results. The output of the proposed method is compared with the output of the conventional two phase position detection method. Even though the proposed method uses the same number of Hall Effect based magnetic position sensors, it produces better position information compared to the conventional two phase system.
